# Effect of Signal Design of Autonomous Vehicle Intention Presentation on Pedestrians’ Cognition

**DOI:** 10.3390/bs12120502

**Published:** 2022-12-08

**Authors:** Chih-Fu Wu, Dan-Dan Xu, Shao-Hsuan Lu, Wen-Chi Chen

**Affiliations:** 1 The Department of Industrial Design, Tatung University, Taipei 104, Taiwan; 2 The Graduate Institute of Design Science, Tatung University, Taipei 104, Taiwan; 3 Bareiss Taiwan Technology Co., Ltd., 28F.-5, No. 93, Sec. 1, Xintai 5th Rd., Xizhi Dist., New Taipei City 22175, Taiwan

**Keywords:** intention presentation of autonomous vehicles, vulnerable pedestrians, connected and autonomous vehicles, signal design, human–vehicle interaction

## Abstract

In this study, a method is devised that allows the intentions of autonomous vehicles to be effectively communicated to pedestrians and passengers via an efficient interactive interface. Visual and auditory factors are used as variables to investigate the effects of different autonomous vehicle signal factors on the judgment of pedestrians and to determine the main factors such that the best combination can be proposed. Two visual dimensions (i.e., color and flashing) and three auditory dimensions (i.e., rhythm, frequency, and melody) are used as the experimental signal variables. In addition, deceleration and waiting-to-restart scenarios are investigated. Multiple-choice questions and a subjective cognition scale are used for evaluation. The results show that the combination of green and slow rhythm can be used for the road-user-first case, whereas the combination of red and fast rhythm can be used for the vehicle-first case. Under the same intention, factors of color, flashing, rhythm, and melody are highly similar in terms of the combination mode, except for the frequency. In the deceleration and waiting-to-restart scenarios, the frequencies of the best signal are high and low frequencies, respectively. The results of this study can be used as a reference for the signal design of autonomous vehicles in the future and provide ideas for the interactions between autonomous vehicles and pedestrians.

## 1. Introduction

Autonomous driving will become one of the mainstream modes of transportation in the future. Consequently, potential traffic safety issues associated with autonomous driving have received significant attention. Some researchers argue that introducing autonomous driving to vehicles may reduce the overall frequency and severity of crashes and personal injuries [1]. However, if misunderstanding occurs in the communication between autonomous vehicles and pedestrians, then undesirable consequences are inevitable [2]. Currently, drivers typically communicate their driving intentions to pedestrians via signals from the vehicle, eye contact, gestures, etc. [3,4,5,6,7]. These communication methods (facial expression, eye contact, gesture, vehicle movement, and the sound from the vehicle) allow pedestrians to clearly understand the intentions of car drivers and to be aware of upcoming vehicles [8,9,10,11,12]. While crossing a road, pedestrians can assess whether they can safely cross an intersection based on the speed and acceleration of vehicles as well as the distance between the vehicles and themselves [13,14]. However, unlike humans, autonomous vehicles have not yet developed the capability to communicate with pedestrians through using and interpreting implicit and explicit communication signals [15]. Furthermore, autonomous vehicles cannot accurately simulate the typical human–vehicle interactions, such as gestures and eye contact [2]. Therefore, traffic accidents and serious injuries may occur when the signals provided by autonomous vehicles to pedestrians and other vehicles are ambiguous. To ensure the safety and acceptance of autonomous vehicles and the trust of other pedestrians, communication strategies between autonomous vehicles and other pedestrians must be developed [16]. These strategies must ensure that autonomous vehicles can interact and communicate with other pedestrians for safer driving. This task is particularly challenging for mixed traffic involving autonomous and manual vehicles [17]. In particular, considering the most recent development of autonomous vehicles, informing about “vehicle behavior” and “vehicle intention” without any direct interaction between the driver and the external environment is becoming increasingly more important [18]. When autonomous vehicles begin to extend from the original restricted areas to open roads with both pedestrians and vehicles, the conventional vehicle indicator light and other typical interaction methods must be adapted appropriately. Researchers have investigated the effect of the intention transmission of autonomous driving from the visual level via lighting, light-emitting diodes (LEDs), or projections [2,14,15,19]. Additionally, researchers have focused on auditory feedback [14,19] and used additional devices [14] or humanoid robots [8] to assist in transmitting vehicle information. Pugliese, Brian J., et al. (2020) [20] reported that pedestrians rely primarily on visual signals to assess the safety risks when crossing a road, whereas they regard auditory signals as auxiliary information. However, a consensus regarding the optimal combination of light and sound for vehicle intention transmission has not been reached. Moreover, the factors of light and sound require further investigation. The optimal combination of light and sound that would result in the highest usability and is most consistent with the cognitive preferences of pedestrians is yet to be identified.

The purpose of this study is to investigate the signal design of autonomous driving based on different scenarios, such that important factors that affect cognition can be identified and a better design combination that is conducive to the cognition of pedestrians can be determined. The findings of this study will serve as a reference for future interactions between pedestrians and autonomous vehicles.

## 2. Literature Review

Unambiguous vehicle signals are key for resolving traffic ambiguity. Inadequate communication or communication errors are likely to cause traffic accidents [21,22,23], whereas unambiguous signals can inform pedestrians of the vehicle’s intentions or provide guidance signals for further action [24]. Research shows that more than a quarter of traffic accidents are caused by ineffective communication between pedestrians and drivers, particularly when pedestrians decide to cross a road and feel insecure owing to insufficient clear signals [25]. Additionally, gender difference among pedestrians is an important factor. Women generally appear to be more cautious than men and more likely to abide by traffic rules [26,27,28,29,30]. Thus, developing an interactive interface for a new traffic environment is difficult as it necessitates user friendliness with the least training. Moreover, the task time should be short and the high cognitive demand low [31]. In particular, when an accident risk is present, the new interactive interface should be easy to use and learn [32,33]. Additionally, users should be able to understand the information and react accordingly in different locations without further training and testing [34,35]

Currently, well-known research teams and automobile companies are investigating the intention communication signals of autonomous vehicles from different aspects. Klaus Bengler et al. (2020) [36] classified existing studies pertaining to external human–machine interfaces (eHMIs) into four categories: (1) classifying vehicles as “autonomous driving”; (2) implementing light strips based on light mode; (3) displaying text and icon information simultaneously; and (4) displaying information on roads via laser projection. Prior studies showed that pedestrians can understand the signals from LEDs in different orders on the top of the windshield of a vehicle after a brief training period, thus allowing communication between vehicles and pedestrians to be established. Signals from LED interfaces were used to replace eye contact between the driver and road user, thus providing a clearer and more prompt communication mode. However, without learning these functions in advance, pedestrians cannot understand the meaning and indications of the signals [37].

Mitsubishi used the direction of road lighting as an indicator. This indicator can project large and easily understood animation lighting on a road surface to indicate the intention of a vehicle, such as driving forward or backward [38]. Semcon installed a display screen in the front of a vehicle and performed smiling as an interface to interact with pedestrians to confirm that the vehicle would stop for them. Research showed that pedestrians felt comfortable crossing the road when such strategies were applied [39]. Additionally, researchers have evaluated the designs of various “walking” and “no walking” signs. The results showed that pedestrians were more likely to decide to cross a road based on certain reference indicators (e.g., the human–vehicle distance and the human–vehicle velocity difference) instead of the information displayed on external displays [15]. Based on the overall preferences of pedestrians, Métayer & Coeugnet (2021) [40] concluded that LED light strips and pictograms were the two most typically used eHMIs. Both were considered visible and easily understood.

Regarding the color of light, Zhang et al. (2017) [41] discovered that drivers of non-autonomous vehicles associate green with moving vehicles and red with decelerating or stopped vehicles. Furthermore, they associate forward lights with moving vehicles and reverse lights with decelerating or stopped vehicles. Bazilinsky et al. (2019) [42] reported that color imposed the most significant effect on users crossing a road (“walking” and “stop”). Pedestrians were the most convinced of the notion of “walking” being indicated by green signals. This feedback was primarily based on the understanding of signal lights by pedestrians [43]. In human–vehicle interactions, the front brake light of an autonomous vehicle should be green, which is similar to the reuse mode of front brake lights adopted by Declercq et al. (2019) [44] and Petzoldt et al. (2018) [19]. Mahadevan et al. (2017) [45] investigated various interaction modes, including audio, video, and animation. In the absence of a clear intention display mechanism, pedestrians decide whether to cross based on the speed and distance of vehicles. In terms of communication methods, pedestrians typically prefer LED sequence signals to liquid-crystal displays (LCDs) and other communication methods, such as auditory and physical prompts. Researchers have suggested the combination of vision, physics, and sound. Additionally, studies have shown that the location of information prompts is unrestricted, i.e., they can be on a vehicle or within a certain environment.

Shuchisnigdha et al. (2018) [46] designed new sound and visual signals for fully autonomous vehicles. Their experimental results showed that, in terms of vision, the silhouettes of words and walking were favored more by the participants than the dynamic display of a smile. In terms of sound, the participants were concerned that music and horn sounds would be interfered with by other sounds in a traffic environment; therefore, they preferred oral information. Merat et al. (2018) [47] discovered that pedestrians preferred light and sound to text and voice when interacting with autonomous vehicles, whereas Chang et al. (2018) [48] believed that the most accurate presentation method for autonomous vehicle information was text. Stadler et al. (2019) [49] and Othersen et al. (2018) [50] showed that the preferred human–vehicle communication was in the form of images. Meanwhile, some researchers suggested that in the future, autonomous vehicles would be able to communicate with pedestrians via social robots installed on the driver’s seat [8]. Other scholars suggested the use of wearable sensors by pedestrians, which will enable the transmission of various warning signals regarding vehicle intentions to other pedestrians [51] (Gordon et al., 2016).

Currently, the design and research of autonomous human–vehicle interactions are still in the development stages. Researchers have not yet determined the features in autonomous human–vehicle interactions that can achieve efficient, accurate, user-friendly communication. Furthermore, the effects of specific parameters of relevant factors have not been discussed adequately [52]. Most studies show that participants benefited significantly from the interactive interface during their interactions with autonomous vehicles. Therefore, the human–vehicle interaction interface and the specifications for autonomous vehicles must be standardized.

## 3. Experimental Design

Based on the characteristics of autonomous vehicles and pedestrians as well as the intention information of autonomous vehicles, we established the control factors of field and intention information required for our experiment. Researchers have extensively investigated vehicle information transmission using various methods during autonomous human–vehicle interactions. The results showed that the location of an additional interactive interface implemented did not impose significant effects. The LEDs of a vehicle can be combined with sound, and different fields can be combined with different autonomous vehicle intentions. In this study, we presented information to participants in the form of films, based on which evaluations were conducted. Next, visual and auditory factors were verified and analyzed. The effects of relevant signal design factors on vehicle intention transmission are discussed. We aim to provide answers to the following questions:

Q1: What are the effects of different combinations of light color and sound of vehicles on the transmission of autonomous driving intentions?

Q2: What is the information combination mode that is the most accepted and understood by pedestrians with different vehicle states and vehicle intentions?

### 3.1. Experimental Sample Setting

The purpose of this study is to determine the relevant factors of human–vehicle interactions and investigate the acceptance of autonomous driving interaction design by pedestrians. To ensure a safe experiment, we performed some tests in advance based on videos [37,48]. A report by the Automotive Research and Test Center stated that LEDs/OLEDs are the current main sources of vehicle light and that they offer rapid operation responses and high luminous efficiency. Furthermore, they provide plasticity and color rendering [53]. Most pedestrians believe that green lights signal an invitation for them to cross a road, whereas red is associated with a warning to stop. In human cognition, red is typically related to “excitement” and “stimulation”, whereas blue and green are related to “safety/comfort” and “gentleness/relaxation”, respectively. The specific design, presentation, and feedback pertaining to the conveyance of vehicle information via light color to pedestrians are yet to be investigated [54]. Traffic signs are designed with red and green lights. The wavelength of red light is longer, and its capability to penetrate air is stronger. Thus, it is more attractive than other colors. Green is used as a contrasting color as it can be easily distinguished from red. Therefore, “red” and “green” were used as visual variables in our experiment. In addition to color, the flashing of light presents certain connotations and inherent meaning in the traffic environment [54]. Flashing can rapidly attract people’s attention [55]. Flashing and flashing modes have been widely used to transmit signals or indicate steering intentions. Flashing headlights are a clear communication tool typically used by drivers when negotiating with pedestrians [54]. Uniform animation (flash and pulse) is more visual and conspicuous than scanning animation (inward scanning, outward scanning, or double scanning) [55].

In terms of sound interaction, researchers have discussed the music, beeping sound, and oral information of “safe crossing” [19]. The psychoacoustic characteristics of sound (rhythm, pitch, and volume) affect people’s psychological perception [56,57,58,59,60]. Similarly, the recognition of approaching objects by people is related to different sound cues [61]. Therefore, sound elements of rhythm, frequency, and melody were used as auditory variables in the current experiment. Single or continuous tone was set for “melody”. The current flashing cycle of traffic signs for pedestrians and car warning lights were set for “rhythm”; two versions of sound rhythm were designed, i.e., fast and slow. The slow version was 0.8 s per time (based on the cycle of traffic signs for pedestrians), and the fast version was 0.5 s per time (which is the fastest cycle of car warning flashing lights). In terms of “frequency”, the original frequency of 500 Hz was changed to 880 Hz to ensure that the sound remained audible in ambient noise [62]. In addition, we found that using a higher frequency (1000 Hz) or a super-high frequency (3000 Hz) can improve the detection rate of sound [63]. Therefore, the sound frequencies used in this study were set to two types, i.e., low and high, whose ranges were 500–880 and 1000–3000 Hz.

**Figure 1:**
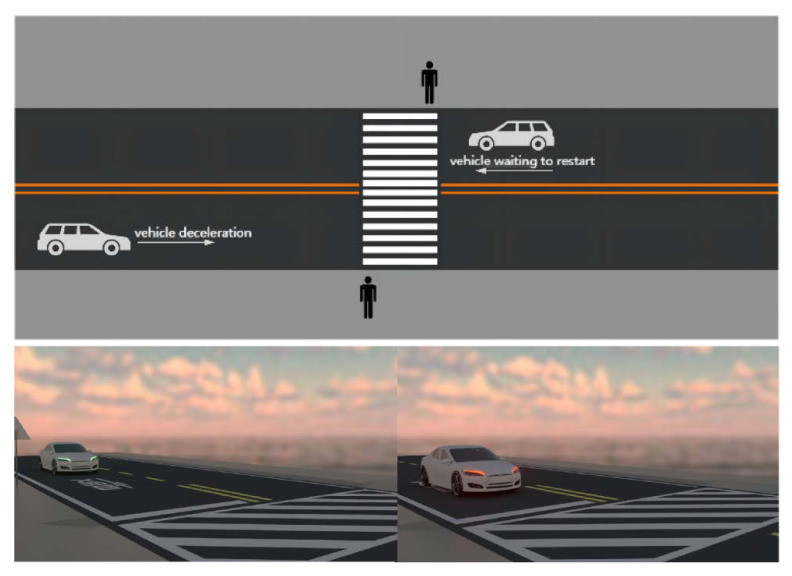
Scene design of animated film.

In the experiment, the scene in the film was set as a vehicle traversing straight in the evening. Two scenarios were investigated, i.e., vehicle deceleration and vehicle waiting to restart. The road featured two-way lanes, zebra crossings, and road user signs. The setting was determined primarily based on the fact that vehicles traversing straight are one of the most typical collision scenarios [64]. Elue et al. (2008) [65] found that pedestrians and vehicles are more likely to have a collision probability during evening or twilight. Therefore, the scene of this study was set at nightfall. The main design parameter was the light brightness in the video, and clouds have no influence on the light brightness. To create a more realistic environment, noise from the road environment was added to the scene in the animated film used in the experiment. Yamauchi et al.’s (2014) [66] study claimed that warning sounds had to be 2–3 dB higher than ambient noise to be detected. Therefore, the sound in the film was adjusted to be 3 dB higher than the ambient noise. This method can ensure the sound detectability for the participants, because the variable of the sound will always be 3 dB higher than the ambient noise. According to the U.K. Department of Transportation (1987), when a vehicle is traversing at 40 mph, the probability of death of pedestrians is 85%. Therefore, the vehicle speed in the video was set to 40 mph. The braking reaction distance was 30 to 35 m, and the reaction time of pedestrians was 2–3 s. The scene design of the animated film is shown in Figure 1.

Based on the shape of existing vehicles, we set the visual variables used in this experiment based on the original signals (light signals). The first visual variable was the light representation, and the second visual variable was the light color. In this experiment, the sound elements (rhythm, frequency, and melody) were regarded as auditory variables. Each of the three elements comprised two variables. “Melody” was classified into single tone and continuous tone. In terms of “rhythm”, the slow version featured 0.8 s per time (the traffic sign cycle of pedestrians), and the fast version featured 0.5 s per time (the fastest cycle of car warning flashing lights). Meanwhile, “frequency” was classified into low and high frequencies, whose ranges were 500–880 and 1000–3000 Hz, respectively. The sound samples were downloaded from the websites of SOUND JAY and freeSFX. The rhythms and frequencies of the sound were edited using Audacity. The specific combinations of variables used are listed in Table 1.

### 3.2. Experimental Procedure

To investigate the key factors in the interaction between autonomous vehicles and pedestrians, we set the headlight color, flashing mode, and sound elements (rhythm, frequency, and melody) of autonomous vehicles as the control variables in the experiment. We used 32 films set for each scenario (the deceleration and waiting-to-restart scenarios), which comprised 64 films. This experiment was conducted in a quiet multimedia lab. The equipment was an Apple MacBook Pro featuring a 16-inch screen and Retina display with a six-speaker sound system and studio-grade microphone. To ensure participants could clearly identify and distinguish the visual and auditory variables in the experiment, they were required to watch the video at a reasonable and comfortable distance. The participant was required to complete the intention judgment 4 s before the film ended. After completing a scenario test, each participant was allowed to rest for 30–60 min before performing another scenario test. To avoid the learning effect between two different scenarios and films, each participant was instructed to conduct two scenario tests with different film starting sequences. After watching each film, the participants were required to select their intention and provide answers to subjective cognitive assessment questionnaires. We investigated the intention selections of each participant for each film based on three options: road user first, vehicle first, and unclear. In particular, the intention judgments by pedestrians were investigated based on their intuitive cognition. Therefore, no correct answer was set for the signal content. The signal intention was determined based on the participants’ judgment. Subsequently, subjective cognitive perception was measured. The items of the questionnaire, i.e., signal usability, signal usefulness, and signal satisfaction, were designed based on the technology acceptance model and the satisfaction degree. Each item was evaluated using the Likert five-point scale. After the questionnaire was completed, a short interview was conducted to understand the feelings and opinions of the participants who watched the film. The scoring items were as follows: The perceptual usability of autonomous vehicle signals was used to evaluate whether the combination of autonomous vehicle signals can be understood easily by pedestrians to determine the vehicle’s intentions. The perceptual usefulness of autonomous vehicle signals was used to evaluate whether the combination of autonomous vehicle signals rendered it safer and more efficient for pedestrians to cross a road. The satisfaction of autonomous vehicle signals was used to evaluate the willingness to use the combination of autonomous vehicle signals for signal transmission.

## 4. Results and Discussion

The independent variables of intention judgment are a combination of five signals, i.e., the color of autonomous vehicle lights, flashing mode, sound rhythm, sound frequency, and sound melody. Two scenarios were investigated, i.e., deceleration and waiting-to-restart scenarios. Each scenario featured 32 signal judgment selections. In total, 35 participants participated in the experiment, and their basic information is shown in Table 2. Participants’ gender, age, and driving experience are at a reasonably level distribution, and they have no color blindness and could clearly watch the experimental video. Thus, the participants are representative of a larger population.

### 4.1. Reliability Analysis of Questionnaire

The answer selected for signal intention judgment was a discontinuous score. The internal consistency reliability was used to test the reliability of the results of the intention questionnaires based on two scenarios. Calculation results show that the Cronbach’s α values of the deceleration and waiting-to-restart scenarios were 0.808 and 0.802, respectively, which indicates high reliability for the subsequent Pearson’s chi-square test. In the test of goodness-of-fit, in the deceleration scenario df = 2, *p* < 0.001, in the waiting-to-restart scenario df = 2, *p* < 0.001, and the expected frequency <5 is 0.0%, indicating that the verification has reached the basic hypothesis of a sufficient sample number. This result indicates that it has reached a sufficient sample number. The subjective evaluation questionnaire was evaluated based on the Likert five-point scale. The Cronbach’s α values of the deceleration and waiting-to-restart scenarios were calculated to be 0.847 and 0.818, respectively, which indicates high reliability for the subsequent multivariate analysis. Through the test of descriptive statistics, it can be found that the skewness and kurtosis values of usability, usefulness, and satisfaction range from 1 to −1, which means the data conform to normal distribution.

### 4.2. Analysis Results of Vehicle Deceleration Scenario

The judgment results of vehicle intention are presented in this section. Based on the ISOTC145/SCI public information symbol design program, the recognition accuracy of a standard image symbol should exceed 67% for mass communication. Therefore, the questionnaires were listed based on the percentage (from high to low) of “road-user first” and “vehicles first”, and 67% was specified as the delineating percentage, as shown in Table 3.

To investigate the factors that affect the judgment of pedestrians in the deceleration scenario, we analyzed the intention options. The chi-square test was performed to test the significance of each factor, and the results show in Table 4 that the flashing mode, sound frequency, and melody did not significantly affect the deceleration scenario. The intention indicated horizontal significance for color (chi = 12.000, *p* = 0.002). Based on the percentage difference, the percentage of “road-user first” selecting green was 100%, and the percentage of “vehicle first” selecting red was 100.00%. The intention was significant for rhythm (chi = 8.867, *p* = 0.012). Based on the percentage difference, the percentage of “road-user first” selecting slow rhythm was 83.33%, and the percentage of “vehicle first” selecting fast rhythm was 100.00%.

The results of multivariate analysis of variance (MANOVA) for the subjective cognitive assessment based on the deceleration scenario are summarized in Table 5. As shown, color, flashing, and melody imposed significant effects, and the combinations of color × rhythm, color × melody, color × flashing × rhythm × melody exerted significant interaction effects.

The effect verification of the combinations is shown in Table 6. As shown, color, color × rhythm, and color × melody significantly affected usability; color, flashing, color × rhythm, and color × melody significantly affected usefulness; and color, flashing, melody, color × rhythm, and color × melody significantly affected satisfaction.

Next, the simple main effect results of items with two-factor interactions are summarized in Table 7. In terms of usability, the averages of green × slow rhythm, red × fast rhythm, and red × single tone were higher than those of other combinations. In terms of usefulness, the averages of green × slow rhythm, red × fast rhythm, and red × single tone were higher than those of other combinations. In terms of satisfaction, the averages of red × fast rhythm and red × single tone were higher than those of other combinations.

Based on the deceleration scenario, factors without two-factor interactions were organized. The descriptive statistical averages are shown in Table 8. The averages of flashing and high frequency in the categories of usability, usefulness, and satisfaction were greater than those of other factors.

Based on the results above, the recommended parameter combinations of the signal factors corresponding to “road-user first” and “vehicle first” in the deceleration scenario are discussed next. Color significantly affected intention discrimination and subjective cognitive assessment. In terms of intention, the percentage of “road-user first” selecting green was 100%, and the percentage of “vehicle first” selecting red was 100.00%. The flashing mode showed no significance in intention discrimination but indicated significance in subjective usefulness and satisfaction. As flashing did not impose a two-factor interaction effect, flashing was selected based on the average value. Most of the participants mentioned that when they noticed the flashing signal of autonomous vehicles, they were able to promptly discern that they were detected by the vehicle system. The constant lighting caused the participants to form incorrect assumptions regarding the light or headlights of the vehicle. Therefore, using the flashing mode in autonomous vehicles for both “road-user first” and “vehicle first” in the deceleration scenario is considered reasonable. Rhythm showed significance in terms of both intention discrimination and subjective satisfaction. In the deceleration scenario, the percentages of pedestrians first selecting slow and fast rhythms were 83.33% and 100.00%, respectively. Based on the two-factor interaction, the signal combination of color and rhythm indicated interaction. The recommended combinations were green * slow rhythm and red * fast rhythm, which were the same as the intention judgment results. The participants mentioned that when they heard a signal with a fast rhythm, the intention of “vehicle first” was clear, whereas they required more time to decipher the intention of a signal with a slow rhythm. Frequency showed no significance in terms of either intention discrimination or subjective satisfaction and indicated no interaction with other factors. High frequencies indicated significance in subjective cognition. In the deceleration scenario, high-frequency signals were more prevalent than low-frequency signals. Melody showed no significance in intention discrimination but indicated significance in subjective satisfaction. Based on the two-factor interaction, the signal combination of color and melody indicated interaction, and the recommended combination was red * single tone. When the melody was assessed based on the intention, the number of “road-user first” was the same for both continuous and single tones. To distinguish “road-user first” from “vehicle first,” the melody was set to a continuous tone.

Based on the results above, the combination of variables conforming to the signal recommendation scheme is shown in Table 9.

### 4.3. Analysis Results of Waiting-to-Restart Scenario

After the questionnaire results for the waiting-to-restart scenario were organized, they were ranked based on the statistical percentage of the signal intention answers. The results shown in Table 10 are based on 67% as the benchmark.

Pearson chi-square test results for waiting-to-restart scenario are shown in Table 11, the flashing mode, sound frequency, and melody were not significant, whereas the color was significant (chi = 8.167, *p* = 0.017). Based on the percentage difference, the percentage of “road-user first” selecting green was 100.00%, and the percentage of “vehicle first” selecting red was 100.00%, which was significantly higher than the average level of 50.00%. In the waiting-to-restart scenario, rhythm showed horizontal significance (chi = 8.167, *p* = 0.017). Based on the percentage difference, the percentage of “road-user first” selecting slow rhythm was 100.00%, and the percentage of “vehicle first” selecting fast rhythm was 100.00%.

The results of MANOVA for the subjective cognitive assessment in the waiting-to-restart scenario are summarized in Table 12. Flashing showed significance, and color * rhythm indicated a significant interaction effect.

The effect verification of the combinations is shown in Table 13. As is shown, flashing and color × rhythm significantly affected usability, usefulness, and satisfaction.

Next, the simple main effect verification results of items with two-factor interactions are summarized in Table 14. In terms of usability, the averages of green × slow rhythm and red × fast rhythm were higher than those of other combinations. In terms of usefulness and satisfaction, the average of red * fast rhythm was higher than those of other combinations.

Factors without two-factor interactions were organized for the waiting-to-restart scenario. The descriptive statistical averages are shown in Table 15. The averages of flashing, low frequency, and single tone in the usability, usefulness, and satisfaction categories were greater than those of other factors.

Based on to the results above, the recommended parameter combinations of the signal factors corresponding to “road-user first” and “vehicle first” in the waiting-to-restart scenario are discussed next. Color significantly affected the intention discrimination. The percentage of “road-user first” selecting green was 100%, and the percentage of “vehicle first” selecting red was 100.00%, which was the same as the results of the deceleration scenario. Most participants mentioned that in the waiting-to-restart scenario, as the autonomous vehicle was stopped, they would assume that the vehicle would yield to the pedestrians. The signal intention presented by the green light further confirmed the “road-user first” intention. When the red light was signaled, the pedestrians were uncertain about the intention conveyed by the signal. Therefore, the judgment of intention was delayed. The flashing mode showed no significance in intention discrimination but indicated significance in subjective cognitive assessment. Since flashing did not impose a two-factor interaction effect, it was selected based on the average value. Rhythm showed significance in intention discrimination. In the waiting-to-restart scenario, the percentage of “road-user first” selecting slow rhythm was 100%, and the percentage of “vehicle first” selecting fast rhythm was 100.00%. Based on the two-factor interaction, the signal combination of color and rhythm indicated interaction. The recommended combinations were green * slow rhythm and red * fast rhythm, which were the same as the intention judgment results. Frequency showed no significance in terms of either intention discrimination or subjective satisfaction and indicated no interaction with other factors. The low frequency was selected based on the average value. The participants mentioned that they preferred the low-frequency sound signal when the vehicle was near them. Melody showed no significance in terms of either intention discrimination or subjective satisfaction and indicated no interaction with other factors. The single tone was selected based on the average value. The recommended combination was reorganized based on the deceleration scenario, and the recommended melody was a continuous tone with the intention of “road-user first”.

Based on the results above, the combination of variables conforming to the signal recommendation scheme is shown in Table 16.

## 5. Conclusions

Owing to the gradual increase in commercialization and marketization, autonomous vehicles have become the future development trend. In this regard, traffic safety is the primary problem to be solved. The interactions between autonomous vehicles and pedestrians must be analyzed and discussed comprehensively. Lights and sound are the main human–vehicle interaction sources. In this study, the different colors and presentation methods of existing lights as well as the rhythm, frequency, and melody of sound were investigated. The results showed that, for different driving motivations, in the deceleration and waiting-to-restart scenarios, light color and sound rhythm were the main factors affecting the road user’s interpretation of intentions. For “road-user first”, green and slow rhythm were selected, and for “vehicles first”, red and fast rhythm were selected. In addition, the results of the subjective cognitive assessment revealed that, among all factors, flashing showed significance in terms of both the deceleration and waiting-to-restart scenarios. As such, flashing was used as the best signal in both scenarios. A comparison between the best signal combinations for the deceleration and waiting-to-restart scenarios showed that except for the frequency, the color, flashing mode, rhythm, and melody were highly similar to those for the same intention judgment. In the deceleration and waiting-to-restart scenarios, the best signals featured high and low frequencies, respectively. The overall results showed that the visual factor was superior to the auditory factor in the deceleration scenario, whereas the visual and auditory factors offered the same advantages in the waiting-to-restart scenario.

The effects of different autonomous vehicle signal factors on the cognition of pedestrians were discussed herein. Based on the different situations encountered by autonomous vehicles, we obtained the best signal combinations of different factors via experiment. In this study, the scene of the video was set in the evening on a road where vehicles were going straight and pedestrians were crossing the road without the aid of traffic lights. This situation makes it difficult for pedestrians to judge whether they should pass, which made the scene prone to collision. By studying pedestrians’ cognitive judgment of self-driving cars’ signals in this situation, the optimal signal combination can be obtained and applied to most general road scenarios. The experiment in this study is reproducible. The independent variables include two visual variables (color and status) and three sound variables (melody, rhythm, and frequency). The design of experimental variables is comprehensive; thus, the proposed design of the signal sets has good credibility and feasibility. With the increasing use of autonomous vehicles, the research can be more applicable for designing the signals for pedestrian and self-driving cars as a reference for future research.

To account for safety, we used animated films to present the scenarios. Therefore, the participants were not instructed to perform a safety risk assessment, which is necessary in the actual situation. As such, the participants’ responses and cognitive evaluations might be affected. Due to the use of video simulation, the study did not consider the distance between vehicles and pedestrians, and differences of the close-distance signal. The purpose of our research is mainly focused on pedestrians’ cognitive judgment of self-driving cars’ signals, which emphasizes the understanding of these signals. The state of these signals can be clearly reflected through the image simulation. The experimental videos can be watched and distinguished by the participants to make further judgments. It is feasible to study the communication intentions between pedestrians and self-driving cars, and the results can be similar to real situations. Based on our experimental findings, VR and other technologies can be used to further investigate and analyze the color and rhythm of autonomous vehicles’ signals, which primarily affect the cognitive judgment of pedestrians on signal intentions. Future autonomous vehicle signal designs are expected to improve the safety and efficiency of users crossing roads.

## Figures and Tables

**Table 1:** Combinations of sound and visual variables.

Interactive Design Elements of Autonomous Driving	Type	Parameter
Lighting	Color	Red
		Green
	Status	Flash
		Always on
	Melody	Single tone
		Continuous tone
Sound	Rhythm	Fast: 0.5 s/time
		Slow: 0.8 s/time
	Frequency	High frequency: 1000–3000 Hz
		Low frequency: 500–880 Hz

**Table 2:** Basic information of the participants.

Item	Option	Number of People	Percentage (%)
Gender	Male	17	48.57
	Female	18	51.43
Age	Youth	17	48.57
	Middle-aged and elderly	18	51.43
Driving experience	No driving experience	7	20.00
	Less than 1 year	6	17.14
	1–3 years	5	14.29
	4–10 years	3	8.57
	More than 10 years	14	40.00
Traffic accident	With accidents	17	48.57
	Without accidents	18	51.43
Role in the accident	Driving	13	37.14
	Motorcycle riders	4	11.43
	No	18	51.43

**Table 3:** Percentage of intention with different variable combinations for road-user first and vehicle first in deceleration scenario.

Road-User First	Color	Lighting	Rhythm	Frequency	Melody	Percentage
video1	Green	Always on	Fast	Low	Single tone	71.4%
video23	Green	Flash	Slow	High	Single tone	71.4%
video27	Green	Flash	Slow	Low	Continuous tone	71.4%
video31	Green	Flash	Slow	High	Continuous tone	71.4%
video21	Green	Always on	Slow	High	Single tone	68.6%
video25	Green	Always on	Slow	Low	Continuous tone	68.6%
Vehicle First	Color	Lighting	Rhythm	Frequency	Melody	Percentage
video26	Red	Flash	Fast	High	Single tone	77.1%
video32	Red	Always on	Fast	Low	Single tone	74.3%
video18	Red	Flash	Fast	High	Continuous tone	68.6%
video30	Red	Flash	Fast	Low	Single tone	68.6%
video24	Red	Always on	Fast	Low	Continuous tone	68.6%
video28	Red	Always on	Fast	High	Single tone	68.6%

**Table 4:** Statistical results of Pearson chi-square test in deceleration scenario.

Item	Value	Deceleration Intention (%)			χ2	*p*
		1.0	2.0	3.0		
Color	Green	6(100.00)	0(0.00)	10(50.00)	12.000	0.002 **
	Red	0(0.00)	6(100.00)	10(50.00)		
Lighting	Always on	3(50.00)	3(50.00)	10(50.00)	0.000	1.000
	Flash	3(50.00)	3(50.00)	10(50.00)		
Rhythm	Fast	1(16.67)	6(100.00)	9(45.00)	8.867	0.012 *
	Slow	5(83.33)	0(0.00)	11(55.00)		
Frequency	Low	3(50.00)	3(50.00)	10(50.00)	0.000	1.000
	High	3(50.00)	3(50.00)	10(50.00)		
Melody	Single tone	3(50.00)	4(66.67)	9(45.00)	0.867	0.648
	Dual tone	3(50.00)	2(33.33)	11(55.00)		

* *p* < 0.05; ** *p* < 0.01.

**Table 5:** MANOVA results of subjective cognition in deceleration scenario.

Effect	Value	F	df	Degree of Freedom Error	Significance	n^2^_p_
Color	0.940	5631.287	3.000	1086.000	0.000 **	0.940
Lighting	0.010	3.477	3.000	1086.000	0.016 **	0.010
Melody	0.010	3.487	3.000	1086.000	0.015 **	0.010
Color × Rhythm	0.008	2.926	3.000	1086.000	0.033 **	0.008
Color × Melody	0.022	8.248	3.000	1086.000	0.000 **	0.022
Color × Lighting × Rhythm × Melody	0.009	3.453	3.000	1086.000	0.016 **	0.009
Melody	0.010	3.487	3.000	1086.000	0.015 **	0.010

** *p* < 0.01.

**Table 6:** Verification and analysis results of effects among different subjective cognitive combinations in deceleration scenario.

Source	Dependent Variable	Type 2 SS	Degree of Freedom	Mean Square	F	Significance	n^2^_p_
Usability	Color	9.844	1	9.844	10.448	0.001 **	0.010
	Color × Rhythm	22.008	1	22.008	23.360	0.000 **	0.021
	Color × Melody	9.108	1	9.108	9.668	0.002 **	0.009
Usefulness	Color	6.758	1	6.758	7.036	0.008 **	0.006
	Lighting	6.758	1	6.758	7.036	0.008 **	0.006
	Color × Rhythm	15.322	1	15.322	15.953	0.000 **	0.014
	Color × Melody	4.251	1	4.251	4.426	0.036 *	0.004
Satisfaction	Color	5.432	1	5.432	5.609	0.018 *	0.005
	Lighting	8.229	1	8.229	8.497	0.004 **	0.008
	Melody	6.604	1	6.604	6.819	0.009 **	0.006
	Color × Rhythm	7.557	1	7.557	7.803	0.005 **	0.007
	Color × Melody	5.157	1	5.157	5.325	0.021 *	0.005

* *p* < 0.05; ** *p* < 0.01.

**Table 7:** Verification and analysis results of simple main effects of subjective cognition in deceleration scenario.

			Type 2 SS	df	MS	F	*p*	Comparison
Usability	Green	Rhythm	10.066	1	10.066	10.149	0.002 *	Fast rhythm (M = 3.30, SD = 1.032) < Slow rhythm (M = 3.57, SD = 0.958)
	Red	Rhythm	10.618	1	10.618	11.267	0.001 *	Fast rhythm (M = 3.75, SD = 0.947) > Slow rhythm (M = 3.47, SD = 0.994)
		Melody	7.529	1	7.529	7.942	0.005 *	Single tone (M = 3.73, SD = 0.973) > Dual tone (M = 3.39, SD = 1.009)
Usefulness	Green	Rhythm	4.596	1	4.596	4.802	0.029 *	Fast rhythm (M = 3.37, SD = 1.026) < Slow rhythm (M = 3.56, SD = 0.928)
	Red	Rhythm	9.529	1	9.529	9.428	0.002 **	Fast rhythm (M = 3.74, SD = 0.994) > Slow rhythm (M = 3.48, SD = 1.013)
		Melody	6.184	1	6.184	6.081	0.014 *	Single tone (M = 3.72, SD = 0.967) > Dual tone (M = 3.50, SD = 1.049)
Satisfaction	Red	Rhythm	5.972	1	5.972	5.805	0.016 *	Fast rhythm (M = 3.58, SD = 1.017) > Slow rhythm (M = 3.37, SD = 1.011)
		Melody	11.472	1	11.472	11.262	0.001 **	Single tone (M = 3.62, SD = 0.972) > Dual tone (M = 3.33, SD = 1.045)

* *p* < 0.05; ** *p* < 0.01.

**Table 8:** Descriptive statistical average of certain factors in deceleration scenario.

			M	SD	N
Usability	Lighting	Always on	3.47	1.027	560
		Flash	3.58	0.941	560
	Frequency	Low	3.49	1.010	560
		High	3.55	0.961	560
Usefulness	Lighting	Always on	3.46	1.044	560
		Flash	3.61	0.928	560
	Frequency	Low	3.49	1.008	560
		High	3.57	0.971	560
Satisfaction	Lighting	Always on	3.31	1.017	560
		Flash	3.48	0.955	560
	Frequency	Low	3.37	0.999	560
		High	3.43	0.980	560

**Table 9:** Signal combination with the highest average of subjective cognitive assessment for deceleration scenario.

Deceleration Scenario	Color	Lighting	Rhythm	Frequency	Melody
Road-user first	Green	Flash	Slow	High	Continuous tone
Vehicle first	Red	Flash	Fast	High	Single tone

**Table 10:** Percentage of intention with different variable combinations for road-user first and vehicle first in waiting-to-restart scenario.

Road-User First	Color	Lighting	Rhythm	Frequency	Melody	Percentage
video27	Green	Flash	Slow	Low	Continuous tone	80.0%
video23	Green	Flash	Slow	High	Single tone	77.1%
video29	Green	Always on	Slow	Low	Single tone	74.3%
video17	Green	Flash	Slow	High	Continuous tone	74.3%
video19	Green	Always on	Slow	High	Continuous tone	68.6%
Vehicle First	Color	Lighting	Rhythm	Frequency	Melody	Percentage
video26	Red	Flash	Fast	High	Continuous tone	68.6%
video32	Red	Flash	Fast	High	Single tone	68.6%
video18	Red	Always on	Fast	Low	Single tone	68.6%

**Table 11:** Pearson chi-square test results for waiting-to-restart scenario.

Item	Value	Intention Judgment (%)			χ2	*p*
		Road-User First	Vehicle First	Not Known		
Color	Green	5(100.00)	0(0.00)	11(45.83)	8.167	0.017 *
	Red	0(0.00)	3(100.00)	13(54.17)		
Lighting	Always on	2(40.00)	1(33.33)	13(54.17)	0.700	0.705
	Flash	3(60.00)	2(66.67)	11(45.83)		
Rhythm	Fast	0(0.00)	3(100.00)	13(54.17)	8.167	0.017 *
	Slow	5(100.00)	0(0.00)	11(45.83)		
Frequency	Low	3(60.00)	1(33.33)	12(50.00)	0.533	0.766
	High	2(40.00)	2(66.67)	12(50.00)		
Melody	Single tone	3(60.00)	2(66.67)	11(45.83)	0.700	0.705
	Continuous tone	2(40.00)	1(33.33)	13(54.17)		

* *p* < 0.05.

**Table 12:** MANOVA analysis results of subjective cognition in waiting-to-restart scenario.

Effect	Value	F	Assumed Degree of Freedom	Degree of Freedom for Error	Significance	Partial Eta Squared
Lighting	0.012	4.571	3.000	1086.000	0.003 **	0.012
Color × Rhythm	0.017	6.279	3.000	1086.000	0.000 **	0.017

** *p* < 0.01.

**Table 13:** Verification and analysis results of effects among subjective cognition combinations in waiting-to-restart scenario.

Source	Dependent Variable	Type III Sum of Squares	Degree of Freedom	Mean Square	F	Significance	Partial Eta Squared
Usability	Lighting	6.451	1	6.451	6.737	0.010 **	0.006
	Color × Rhythm	17.251	1	17.251	18.015	0.000 **	0.016
Usefulness	Lighting	8.575	1	8.575	9.368	0.002 **	0.009
	Color × Rhythm	14.629	1	14.629	15.981	0.000 **	0.014
Satisfaction	Lighting	13.289	1	13.289	12.788	0.000 **	0.012
	Color × Rhythm	11.604	1	11.604	11.166	0.001 **	0.010

** *p* < 0.01.

**Table 14:** Verification and analysis results of simple main effect of subjective cognition in waiting-to-restart scenario.

				df	MS	F	*p*	Comparison
Usability	Green	Rhythm	4.971	1	4.971	5.331	0.021 *	Fast rhythm (M = 3.58, SD = 1.031) < Slow rhythm (M = 3.78, SD = 0.895)
	Red	Rhythm	10.618	1	10.618	10.473	0.001 **	Fast rhythm (M = 3.72, SD = 0.988) > Slow rhythm (M = 3.44, SD = 1.016)
Usefulness	Red	Rhythm	11.184	1	11.184	11.449	0.001 **	Fast rhythm (M = 3.74, SD = 0.983) > Slow rhythm (M = 3.46, SD = 0.993)
Satisfaction	Red	Rhythm	5.765	1	5.765	5.163	0.023 *	Fast rhythm (M = 3.56, SD = 1.061) > Slow rhythm (M = 3.35, SD = 1.052)

* *p* < 0.05; ** *p* < 0.01.

**Table 15:** Descriptive statistical averages of factors in waiting-to-restart scenario.

			M	SD	N
Usability	Lighting	Always on	3.55	1.012	560
		Flash	3.70	0.953	560
	Frequency	Low	3.67	0.962	560
		High	3.58	1.007	560
	Melody	Single tone	3.68	0.959	560
		Dual tone	3.58	1.009	560
Usefulness	Lighting	Always on	3.55	0.971	560
		Flash	3.73	0.947	560
	Frequency	Low	3.68	0.935	560
		High	3.60	0.989	560
	Melody	Single tone	3.68	0.920	560
		Dual tone	3.60	1.003	560
Satisfaction	Lighting	Always on	3.37	1.040	560
		Flash	3.59	0.993	560
	Frequency	Low	3.51	1.015	560
		High	3.45	1.030	560
	Melody	Single tone	3.51	0.997	560
		Dual tone	3.45	1.047	560

**Table 16:** Signal combination with the highest average of subjective cognitive assessment based on waiting-to-restart scenario.

Waiting-to-Restart Scenario	Color	Lighting	Rhythm	Frequency	Melody
Road-user first	Green	Flash	Slow	Low	Continuous tone
Vehicle first	Red	Flash	Fast	Low	Single tone

## Data Availability

The data presented in this study are available on request from the corresponding author. The data are not publicly available due to the relevant data involving the internal information of the institution and personal information, and there being a need for confidentiality.
